# A Review: Inductively Coupled Plasma Reactive Ion Etching of Silicon Carbide

**DOI:** 10.3390/ma15010123

**Published:** 2021-12-24

**Authors:** Katarzyna Racka-Szmidt, Bartłomiej Stonio, Jarosław Żelazko, Maciej Filipiak, Mariusz Sochacki

**Affiliations:** 1 Łukasiewicz Research Network—Institute of Microelectronics and Photonics, Al. Lotników 32/46, 02-668 Warsaw, Poland; Jaroslaw.Zelazko@imif.lukasiewicz.gov.pl; 2 Institute of Microelectronics and Optoelectronics, Warsaw University of Technology, 75 Koszykowa Str., 00-662 Warsaw, Poland; Bartlomiej.Stonio@pw.edu.pl (B.S.); Mariusz.Sochacki@pw.edu.pl (M.S.); 3 Center for Advanced Materials and Technology CEZAMAT, Warsaw University of Technology, 19 Poleczki Str., 02-822 Warsaw, Poland; Maciej.Filipiak@pw.edu.pl

**Keywords:** plasma etching, reactive ion etching, ICP-RIE, silicon carbide, SF_6_, Cr mask, selectivity

## Abstract

The inductively coupled plasma reactive ion etching (ICP-RIE) is a selective dry etching method used in fabrication technology of various semiconductor devices. The etching is used to form non-planar microstructures—trenches or mesa structures, and tilted sidewalls with a controlled angle. The ICP-RIE method combining a high finishing accuracy and reproducibility is excellent for etching hard materials, such as SiC, GaN or diamond. The paper presents a review of silicon carbide etching—principles of the ICP-RIE method, the results of SiC etching and undesired phenomena of the ICP-RIE process are presented. The article includes SEM photos and experimental results obtained from different ICP-RIE processes. The influence of O_2_ addition to the SF_6_ plasma as well as the change of both RIE and ICP power on the etching rate of the Cr mask used in processes and on the selectivity of SiC/Cr etching are reported for the first time. SiC is an attractive semiconductor with many excellent properties, that can bring huge potential benefits thorough advances in submicron semiconductor processing technology. Recently, there has been an interest in SiC due to its potential wide application in power electronics, in particular in automotive, renewable energy and rail transport.

## 1. Introduction

Silicon carbide (SiC) is a prosperous material for different electronic and photonic applications. The physico-chemical properties of SiC, such as high thermal conductivity, resistance to high temperatures, high breakdown voltage, high hardness and high chemical resistance, make it an attractive material for high-power, high-frequency or high temperature semiconductor devices. These features, in combination with SiC polytypism, make SiC an attractive material for optoelectronic applications due to the refractive index specific to a given SiC polytype. The complex microsystems, including MEMS (Micro-Electro-Mechanical Systems) or MOEMS (Micro-Opto-Electro-Mechanical Systems) based on SiC can be used, among others, in pressure sensors, bolometers, microresonators, micromotors, fuel atomizers, gas turbine rotors, UV radiation sensors, high-temperature sensors, and sensors for chemically aggressive environments ([1] and Refs therein). SiC is also used in the production of energy and communication components. According to the “Digitimes Research” report [2], SiC components have a great potential in electric power supplies, especially in high-power ones, and by 2025 they will be responsible for 25% of power semiconductors used in the automotive industry, as SiC components are used, for example, in the construction of electric vehicles (EV). By 2025, the total global sales of EVs are projected to reach 10 million units, accompanied by an increase in demand for SiC-based drive components [2]. It is assumed that in addition to automotive applications, there will be a significant increase in the use of SiC components in photovoltaics, vehicle charging infrastructure and rail transport [3]. Currently, however, the main problems are the high cost of these components and their limited availability.

Although the inductively coupled plasma reactive ion etching (ICP-RIE) method has been known for many years, it is still under development and refinement in terms of etching of various bulk materials, layers or complex multilayer structures. The method is used, for example, for the production of electronic and photonic devices. It is a dry etching method carried out in the plasma of chemically active gases and/or noble gases and enables a selective etching of: metals, semiconductors, polymers, dielectrics, oxide and nitride materials, as well as photosensitive organic materials (photoresists) [4,5,6,7]. The plasma etching method is commonly used in the etching processes of materials characterized by high hardness and chemical inertness, e.g., SiC, GaN, diamond or cubic boron nitride (c-BN) [4].

The ICP-RIE etching is used in processes of a SiC surface preparation (cleaning and “smoothing”), in the production of Schottky diodes [8,9], as well as in certain stages of MEMS production [10]. It is worth mentioning that plasma etching techniques such as CCP-RIE (capacitively coupled reactive ion etching) or ECR-RIE (electron cyclotron resonance-driven reactive ion etching) can also be applied to a controlled modification of surface roughness (including sidewalls surfaces) of semiconductor devices, e.g., GaN/InGaN LEDs on SiC substrates [11]. The ICP system can generate a high-density plasma much more easily under low pressure (~0.2–10 Pa) than CCP-RIE (9–100 Pa), and its density (~10^16^–10^17^ m^−3^) becomes over an order of magnitude higher than that of the CCP-RIE. In turn, the area of operating pressure vs. plasma density for ICP and ECR-RIE is common to some extent, i.e., in the plasma density range of 10^16^–10^18^ m^−3^, but the ICP can generate a high-density plasma under higher pressure (up to 10 Pa) than the ECR-RIE.

Obtaining the appropriate roughness as a result of the microstructure produced is to increase the luminous efficiency of such LEDs [11]. The efficiency increase by roughness is connected with the generation of many scattering obstacles (i.e., active sites) by attack of the plasma that are randomly generated in their spacious extent and configuration. By this, preferred directions with glancing angles are avoided. The deliberate increase of surface roughness is used not only in the construction of light-emitting diodes or solar cells, but also in biomedical devices. Rough surfaces increase the efficiency of optoelectronic devices and develop (enlarge) the surface area for chemical reactions [12].

The plasma etching can be one of the stages in the process of heterostructures fabrication, such as SiC/SiO_2_, SiC/Si and SiC/glass, by direct adhesive bonding and annealing at *T* ≤ 200 °C, for potential electronic, optical, mechanical and biomedical applications [13]. ICP-RIE serves also to obtain: MESA structures of TLM layout in the production of ohmic contacts for SiC [14,15], MOS structures (metal-oxide-semiconductor structures) [16,17], MOSFETs (metal-oxide-semiconductor field effect transistors) [8,18], SiC nanostructures [4,19,20,21] and photoconductive semiconductor switches (PCSs) [22].

This article is a review on etching of SiC using inductively coupled plasma. The ICP-RIE is still the only technique that enables reproducible etching processes with high anisotropy, selectivity and etching rate. This article presents the principles of the ICP-RIE method and representative results of SiC etching. Moreover, undesired phenomena of the ICP-RIE process were indicated, which should be avoided in technology of semiconductor devices. The article is enriched with our SEM photos and experimental results obtained from various ICP-RIE processes.

The samples used in this study were commercial 4H-SiC wafers with polished Si-face. An ICP reactor “PlasmaPro100” (Oxford Instruments Ltd.) equipped with two 13.56 MHz RF power supplies was used. The experiments were carried out on a lower (sample) 6-inch electrode covered with a Si/SiO_2_ wafer. The etching characteristics were studied depending on RIE source power (25–300 W), ICP source power (1750–2500 W) and total gas flow, with chamber pressure fixed at 7 mTorr or 10 mTorr. Most of the experiments were performed using Cr (~0.1–0.3 µm) etch masks, which were deposited by RF magnetron sputtering. The effect of O_2_ addition to the SF_6_ plasma was also investigated to improve the etching rate and/or selectivity.

Scanning electron microscopy (ZISS AURIGA 60) and contact stylus profilometry (“Dektak 150 Surface Profiler”, Veeco Instrument) were used to measure the etching profiles, surface morphology and selectivity.

## 2. The ICP-RIE Method

The ICP-RIE method offers etching conditions to obtain deep anisotropic, isotropic or directional etching of patterns. A compromise between the etching rate and anisotropy can be achieved by modifying of the etching conditions, for example by changing the composition of the working gas, appropriate selection of process parameters or the mask material selection [4]. Etching of 3D spatial structures with the ICP-RIE method allows for obtaining walls of etched patterns with different slopes, which is important in the production of structures for various electronic applications, e.g., PIN diodes, Schottky diodes, avalanche photodiodes, MOSFETs (metal-oxide-semiconductor field effect transistors), JFETs (junction field effect transistors), SITs (static inductive transistors), as well as in MMICs (monolithic microwave integrated circuits).

**Figure 1:**
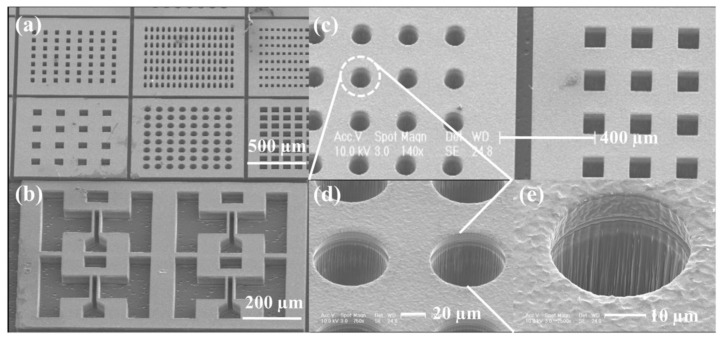
SEM images of various patterns formed on the SiC substrate by the ICP-RIE method: (**a**) Results of etching with using the masks with a different opening width: 35 µm, 25 µm, 20 µm, 100 µm, 70 µm and 70 µm (from the top left, row first); (**b**) vertically etched SiC with a well-etched sidewall profile; (**c**) circular and square etching patterns obtained for the mask opening width of 70 µm. Circular pattern enlarged five and ten times—in (**d**,**e**), respectively. Reprinted with permission from Ref. [23]. Copyright 2021 *Sci. Rep*.

The ICP-RIE etching technology of structures entails the need to produce patterns in the form of vertical, smooth walls forming: holes, deep and narrow trenches or islands [23,24]—the so-called MESA structures (exemplary various etched patterns are shown in Figure 1). Etched parts are used, among others, for interconnections and insulation in integrated circuits, diffractive structures in optoelectronic systems, or for creation of complex MEMS. Large-angle bevel structures (40°–80°) are applicable for diodes, transistors and switches, while small-angle bevel structures (optimally ~7°) are used in APDs (avalanche photodiodes) [23].

One of the highest aspect ratio (18.5:1) trench has been presented by Dowling et al. [25], where the effect of ICP etch parameters on the etch rate and trench profiles has been investigated using popular Ni masking. RIE process itself is also very useful for high-aspect-ratio profiles. Recent advances in reactive ion etching for application in high-aspect-ratio microfabrication have been presented by Huff [26].

### 2.1. Principles of the ICP-RIE Method

The ICP-RIE method uses the phenomena occurring in the plasma and the interaction of ions with the etched material. Reactive plasma is created as a result of a glow discharge in the etching gas (plasma excitation is initiated by an electromagnetic field oscillating usually at a frequency of 13.56 MHz [27]), as a consequence of collisions of electrons, accelerated in the electric field with electrically neutral atoms. Plasma consists of chemically active radicals (uncharged atoms or molecules), ionized atoms, excited atoms, undissociated atoms (molecules) and free electrons.

The main mechanisms of interaction with the removed material in the ICP-RIE are chemical and physical ones [4]. The chemical mechanism is based on the reaction of free radicals with the surface of the etched material to produce of volatile products of this reaction that are pumped out of the reactor. The physical mechanism is based on the high energy ion bombardment and knocking out atoms or agglomerates of atoms of the etched material [4,27].

One of the basic parameters of the ICP-RIE process is the selectivity of etching, defined as the quotient of the etching rate of different materials in the same process, e.g., *V*_SiC_:*V*_Ni_ = 100:12 (for SF_6_ plasma etching [1]), where: *V*_SiC_—the etching rate of SiC; *V*_Ni_—the etching rate of the nickel mask used in the process. A high selectivity etching is required, e.g., in the production of MEMS or MOEMS spatial structures.

The ICP-RIE is a dry etching process that is generally less selective than the wet etching process carried out in aqueous acid and lye solutions. This is due to the participation of two mechanisms in the ICP-RIE process—physical and chemical one, as mentioned above. In the ICP-RIE process, the balance between physical and chemical etching can be controlled by the etching parameters, e.g., plasma gas composition, pressure, temperature of the etched sample, supplied power, and by utilization of mask. The masking material should be selected by analyzing both chemical and sputtering processes in order to avoid a possible micromasking effect.

As for the metal mask, the examples in the literature show that the most promising mask in terms of high selectivity and anisotropy of etched profiles is a mask made of copper. It has been observed that the etching rate of a copper mask in SF_6_ plasma is the lowest compared to the etching rate of other metallic masks, e.g., aluminum or nickel [28]. In turn, the nickel mask is better than the chrome mask, as shown by the results of SiC etching in the SF_6_/O_2_ plasma [23]. The selectivity obtained in etching processes with the use of both masks were: 100:1—for SiC with the Ni mask, and 40:1—for SiC with the Cr mask. The use of non-metallic masks, including photoresist or SiO_2_ can be useful in shallow etching, where patterns with small inclination walls (~45°–50°) can be obtained [29,30]. In the case of the SiO_2_ mask, after the etching of trench structures in the SF_6_/O_2_ plasma, a SiC surface with carbon clusters and non-volatile CF*_x_* compounds was observed [17]. This led to the conclusion that the SiO_2_ mask was poorly suited for ICP-RIE etching of SiC structures.

The second important parameter of the ICP-RIE process is an etching directivity [31], defined by the so-called anisotropy factor *A*, expressed by the Formula (1):

(1) $$A=1-\frac{v_{s}}{v_{p}}$$

where *v_s_*—is the etching rate in the direction tangent to the etched surface, and *v_p_*—is the etching rate in the perpendicular direction. In the case of an isotropic etching, the etching rates in both directions are comparable. A dry plasma etching is isotropic, unless a polymer that blocks the chemical removal of material (the so-called inhibitor) is deposited on the walls of the pattern, e.g., in the plasma process commonly known as the Bosch process, consisting of repeated sequences of etching and passivation steps [4]. In case of a dry anisotropic etching, where the tangential etching rate is close to zero, it is possible to obtain almost vertical MESA structures, i.e., island-shaped structures.

### 2.2. Description of the ICP-RIE Reactor

**Figure 2:**
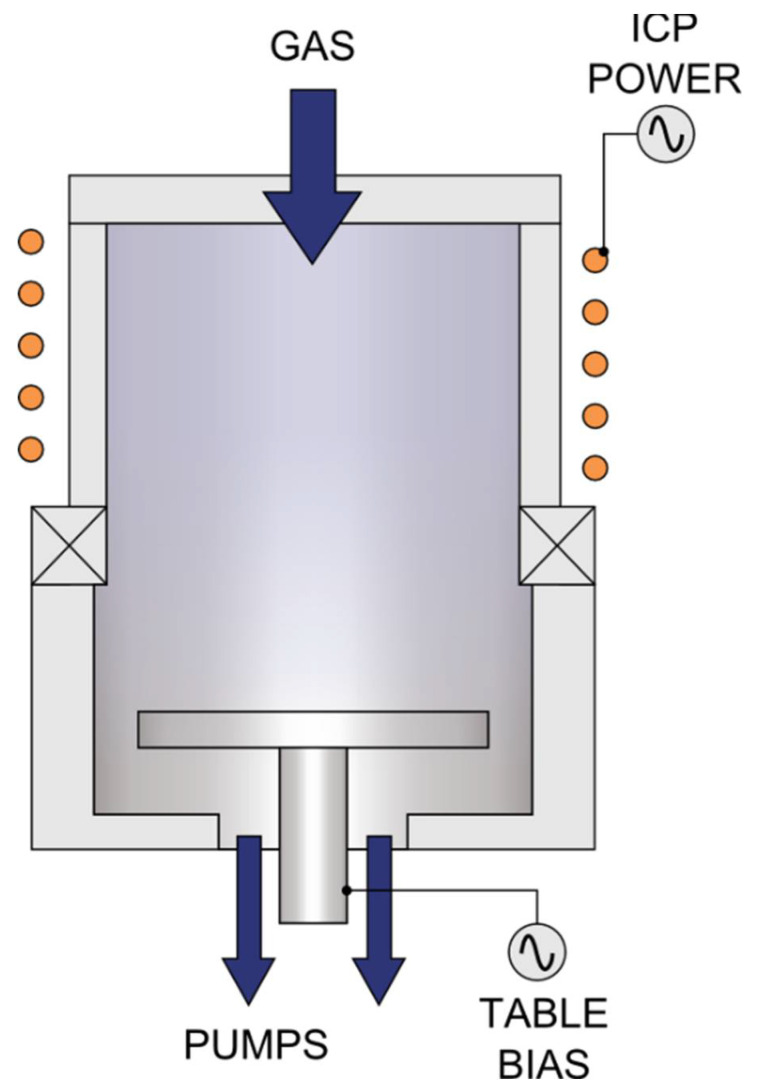
The scheme of the ICP-RIE reactor. Reprinted from Ref. [36].

The cathode powered by the RF generator (13.56 MHz) in the ICP-RIE reactor (in Figure 2) has a smaller surface area than the anode (a lower electrode). The coil placed in the reactor generates a magnetic field that narrows the area of the plasma generated by the RF source, preventing electrons from scattering on walls of the chamber [7]. The etched material is placed on a holder—the lower electrode powered by a second RF generator, which attracts and accelerates the ions from the plasma. The etched material has a negative plasma potential, with an absolute value of up to several hundred volts. It is a constant component of the electrode voltage, induced in the high-frequency glow discharge and the so-called DC self-bias voltage (*U_SB_*). The DC self-bias voltage together with plasma potential (*U_PP_*) are in turn the DC bias voltage (*U_DC_*) [32], as it is expressed by the Formula (2):

(2) $$U_{DC}=\left(-U_{SB}+U_{PP}\right)$$

The large electric field generated near the electrode surface causes a significant acceleration of the ions in the area of its interaction. The ions can obtain energies above 50 eV.

The ICP-RIE reactor enables the RF power control of both RF generators. The RF power affecting the plasma flux is called the inductive power (*P*_ICP_), and the RF power to accelerate the plasma ions, and generate the polarization voltage of the etched material is called RF bias power (*P*_RIE_). The power delivered from both sources influences the etching rate [22,28,33]. Theoretical considerations [34] showed that regardless of the type of etched material and used working gases, the etching rate in the ICP method is directly related to the self-polarization voltage induced by RF power. This was also confirmed by the experimental works, e.g., on SiC [22,28,33,35], in which the increase in the DC bias voltage increases the etching rate.

## 3. Etching of SiC with Different Plasmas

Due to the high chemical resistance of SiC (as a result of a strong covalent bond between Si and C atoms), the ICP-RIE dry etching method is basically the only one that allows obtaining patterns in this material [7]. In the literature, various fluoride plasmas have been used in the ICP-RIE processes of SiC, and also mixtures of fluoride gases with oxygen, argon or helium (see Table 1). There are also examples of the use of other plasmas, e.g., based on chlorine (in Table 1). Fluoride plasma etching processes allow for higher etching rates compared to chloride plasma etching processes [6,23], hence the vast majority of scientific papers concern the use of fluorine-based plasma in ICP-RIE processes.

The plasma etching processes presented in the literature were aimed at optimizing the conditions allowing for increasing the etching rate and obtaining, from the point of view of the subsequent production of the device or the microsystem structure, the “favorable” surface of the etched SiC. In the case of the surface, it was important to avoid damage after etching and also to keep the chemical composition unchanged (i.e., to avoid chemical residues after etching). In general, smooth surfaces increase the performance of microelectronic devices, therefore, after etching, the surface roughness should be kept at a minimum [12]. A low-pressure plasma etching process (~4 mTorr) with a variation in the bias power at fixed plasma conditions can benefit from more anisotropic etch profiles and less plasma damages to the material, which means practically constant surface roughness for different bias power [32].

Examples in the literature [38,42,43] showed that residues on the SiC surface after etching, including fluorine may affect the properties of the produced electronic devices, e.g., Schottky diodes and others. Jiang et al. [42] discussed the possibility of the formation of various types of C-F chemical bonds on the SiC surface: ionic, semi-ionic (i.e., chemical bonds that are neither completely ionic nor completely covalent) and covalent ones. It has been observed that the content of C-F covalent bonds increases with RF power and etching rate. This changes the electrical properties of the surface which becomes less conductive as the etching rate increases.

Choi et al. [54] showed that the structural quality of SiC samples had the negligible effect on results of plasma etching. For SiC samples with different average concentration of standard defects in the form of micropipies, similar etching rates were obtained and similar shapes of the etched MESA profiles were observed. Moreover, the surface roughness of the individual samples measured by AFM (Atomic Force Microscopy) before and after the etching process and expressed by RMS value (root–mean–square of the surface) practically did not differ from each other.

### 3.1. The Influence of Etching Parameters on SiC Surface Morphology, Etching Rate and Angle Profile

In the literature, the influence of the etching process parameters, for example: plasma composition (change in gas mixture composition), gas flow, pressure in the reactor chamber, distance between electrodes, the applied RF power or temperature on the results of the etching process has been investigated. Among others, the influence of these parameters on the etching rate of SiC and on the SiC surface morphology has been studied (see Table 1). It has been established that the etching rate of SiC also depends on the temperature of the substrate holder and reaches its maximum (1.28 μm/min) at temperatures close to 150 °C [45]. Attempts were also made to increase the rate of SiC etching by the initial modification of the studied samples by irradiation them with a laser prior to the etching process. Huang et al. [46] obtained an etching rate of 9365 Å/min, which was increased by 117.18% over the etching rate of the untreated sample, after irradiation of 6H-SiC samples with 800 nm-femtosecond laser at a pulse duration of 120 fs and repetition frequency of 1 kHz. The presence of the SiO_2_ top layer and the increased surface roughness resulting from the modification of samples’ surfaces with the laser beam could contribute to a higher etching rate.

The surface quality is an important factor in technology of electronic devices [5,8,36], and its roughness has a significant influence, among others, on the channel mobility of the MOS transistor [8,58]. It is also worth mentioning that the plasma etching process itself may be one of factors influencing the surface current in p-i-n SiC diodes with MESA structure, which in turn determines the leakage current of these structures [59].

The experimental results of the SiC etching process in C_2_F_6_ + O_2_ plasma indicate a close correlation between the surface morphology of the etched SiC and (*U_DC_*) [32]. It was observed that the roughness of the surface obtained in etching process increases when the distance between the plasma source and the etched sample is reduced, and the power of the RF generator as well as *U_DC_* are increased [32]. The surface roughness is also clearly influenced by the temperature of the substrate during etching process. It was shown that change in the temperature of the substrate holder in the range from 100 to 300 °C can lead to sharp decrease in the root mean square roughness from 153 to 0.7 nm [45].

In the ICP-RIE processes based on fluorinated gas and oxygen the following chemical reactions (3)–(6) are involved:

Si + *x*F → SiF*_x_*,      *x* = 1–4 (3)

C + *x*F → CF*_x_* (4)

C + *y*O → CO*_y_*,      *y* = 1–2 (5)

SiC + *x*F + *y*O → SiF*_x_* + CF*_x_* + CO*_y_* (6)

where F atoms are the main etchant species reacting with Si and/or C atoms, either through pure chemical reaction, or ion assisted chemical reaction or both [49].

Jiang et al. [44] and Osipov et al. [48] observed that the addition of Ar (optimally ~30%) to the SF_6_ + O_2_ gas mixture allows to obtain smoother etched surfaces, as well as increases the etching rate (up to ~500 nm/min), in relation to SiC etched without argon. Moreover, at fixed plasma conditions, the etching rate was also increased with increasing RF power and pressure in the reactor chamber. As the gas flow was increased, the etching rate was initially increased and then decreased. This was explained by the chemical interaction mechanism, in which the different flow rate of the gas used in the process affects the residence time of radicals on the surface of the etched SiC, and it also affects the speed of removal of the resulting volatile reaction products. Faster gas flow helps to remove volatile SiF*_x_* and CF*_x_* compounds faster, however, until a certain optimal flow rate is exceeded (40 cm^3^/min SF_6_, 10 cm^3^/min O_2_ and 20 cm^3^/min Ar), i.e., when the duration of stay of radicals on the surface is shorter than the time needed for a chemical reaction in etching process. It was also noted that the addition of Ar to the SF_6_ + O_2_ plasma had no significant effect on the etching profiles of SiC structures. It is worth mentioning that the SiC etching in a pure Ar atmosphere allows for very low etching rates, e.g., 19 nm/min—for RF power of 200 W and pressure in the chamber of 30 mTorr [37].

It was experimentally established that the dependance of the etching rate of silicon carbide on the percentage of oxygen in the total gas mixture is non-linear [47]. It was observed that the addition of 20–25% oxygen for SiC etching in SF_6_ + O_2_ plasma is optimal for the highest etching rates [23,42,47]. In the process of chemical interaction of reactive fluorine ions with silicon, volatile SiF*_x_* (1 < *x* < 4) compounds are formed, and an addition of the oxygen to plasma increases the etching rate of the carbon-rich SiC surface layer as a result of the formation of volatile compounds in the form of CO, CO_2_ [42] and COF_2_ [39]. The presence of such a layer was confirmed in Auger electron spectroscopy (AES) studies for SiC etched by RIE method in various fluoride plasmas with addition of the oxygen (CBrF_3_ + O_2_, CHF_3_ + O_2_), including the SF_6_ + O_2_ plasma [35,41]. The carbon-rich surface layer has been found to constitute a “blocking layer” potentially limiting the etching rate [35,41]. This layer can be reduced by increasing the oxygen concentration and increasing *U_DC_*. In the papers [35,41] a combined physical and chemical etching model of SiC was proposed. In the first regime, the physical mechanism dominates, and the etching rate is influenced by *U_DC_*, while in the second one (after exceeding a certain critical value of DC bias voltage), the chemical mechanism dominates, and the etching rate depends mainly on the concentration of fluorine and oxygen, and on their reaction with silicon and carbon.

The same conclusion regarding the optimal amount of oxygen—20%—allowing for the highest SiC etching rate was made after the SiC etching process in CHF_3_ + O_2_ plasma [49]. Xia et al. [49] observed an increase in etching rate with increasing oxygen content, then its maximum value (35 nm/min) for 20% O_2_, and after that a sharp decrease almost to zero for 80% O_2_. The initial increase in the etching rate was due to the increase in oxygen addition, which also led to the increase in the number of broken C-F bonds. Consequently, the proportion of reactive F atoms responsible for etching process was increased. The subsequent decrease in the etching rate (with the amount of oxygen above 20%) could be related to the progressive (as the oxygen content increases) effect of “diluting” the concentration of F atoms in oxygen, as well as the possible formation of the SiO*_x_* type oxide layer on the etched surface, which acts as inhibitor [42,49]. Moreover, the authors of the paper [49] noticed that the increase in the amount of oxygen in the plasma deteriorates the quality of the etched surface. This was confirmed by the obtained values of the roughness coefficient (*R_RMS_*) where with increasing O_2_ fraction from 0% to 80%, there was an increase in *R_RMS_* from 1.31 to 2.34 nm. An increase in the SiC surface roughness along with an increase in oxygen content was also observed after etching processes in the Cl_2_ + O_2_ plasma [23]. The increase in SiC surface roughness may be related to the increased efficiency of the oxidation process along with the increase of the oxygen content in plasma [23,49]. Sung et al. [23] also noticed that in some situations the predominance of the oxygen in plasma may contribute to the improvement of the smoothness of the etched surface, e.g., it can remove the micromask (see phenomenon in Section 4.2) formed on the SiC surface by the erosion of the metal mask used in the process.

Due to the appropriate selection of the process parameters, including energy of the bombarding ion beam, it is possible to control the slope of side walls of the etched profiles [5,55]. Kim et al. [55] investigated the ICP-RIE process of SiC structures with a nickel mask etched in the NF_3_ + CH_4_ plasma. They determined the influence of the source power (acceleration of reactive ions), the so-called RF bias power, on *U_DC_* and the angle of side walls inclination (the so-called profile angle) of the etched profiles. The obtained experimental results allowed to conclude on the linear relationship between RF bias power and *U_DC_*, and that increasing the ion bombardment energy leads to decrease in the angle of side walls inclination. Additionally, reducing the angle is associated with an increase in the faceting of the mask, i.e., the increase of its flat surface area.

Obtaining the profiles with different wall inclination angles is also possible by the selection of the appropriate composition of the plasma used in the etching process [22,29]. Sung et al. [23] carried out a detailed analysis of the influence of various plasmas (as gas mixtures) on the obtained profiles, for which it was possible to obtain almost vertical walls, with an inclination angle of 87°. The SF_6_ + O_2_ plasma proved to be the most promising for etching almost vertical profiles and to achieve the highest etching rate of 3050 Å/min. The use of BCl_3_ plasma with addition of nitrogen (BCl_3_ + N_2_), for appropriately selected proportions of these gases, made it possible to obtain structures with an inclination angle ranging from 40° to 80°. When the Cl_2_ + O_2_ plasma was used (with different oxygen fraction), a smaller angle of inclination of the walls was obtained ranging from 7° to 17°.

## 4. Undesired Phenomena of the ICP-RIE Process

### 4.1. Trenching and Microtrenching Phenomena

**Figure 3:**
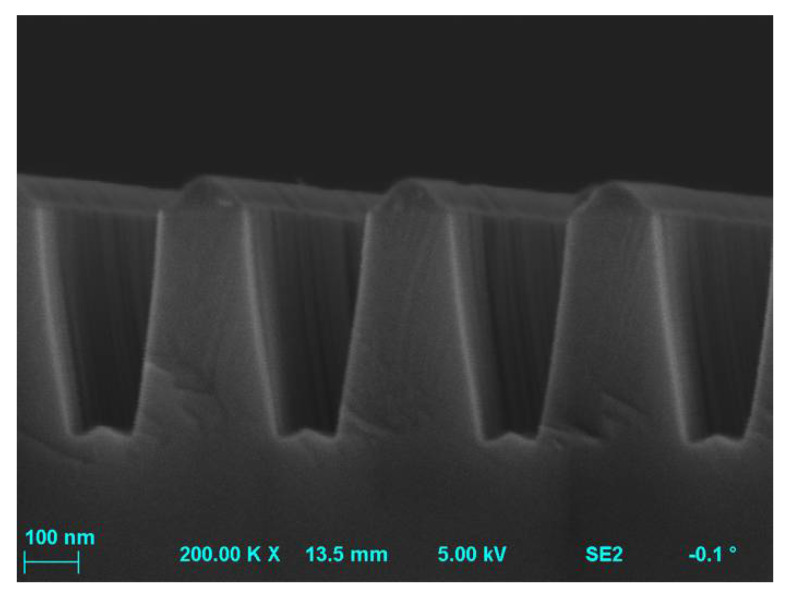
SEM image of etching into SiC—it is visible the trench structure with a period of 360 nm and the etching depth of 430 nm, and the shape determined by a perpendicular incident beam of reactive ions and radicals. The etching process parameters were: O_2_ flow rate = 2 sccm, SF_6_ flow rate = 20 sccm, *P_RIE_* = 100 W, *P_ICP_* = 1300 W, *p* = 10 mTorr, *t* = 10 min.

It was found that the addition of oxygen to the SF_6_ plasma may contribute to the formation of unevenness in the form of trenches at the bottom of the SiC etched profiles. This unevenness arises as a result of secondary etching, which takes place with the participation of ions reflected from the side walls surfaces ([39] and Refs therein). Oxygen-based etching can form a SiF*_x_*O*_y_* layer, which has a greater tendency to accumulate an electric charge (and charge itself) compared to SiC. The increased concentration of ions (attracted by the charged SiF*_x_*O*_y_* layer) on the surface between side walls of the etched profile, as well as an increased proportion of ions reflected from surfaces of these walls leads to an increase of the rate of chemical reactions affecting the formation of a trench structure. The structure of the trench itself may additionally contain heterogeneity (Figure 3) depending on the incidence angle (angular distribution) of the beam of reactive ions and radicals [39].

**Figure 4:**
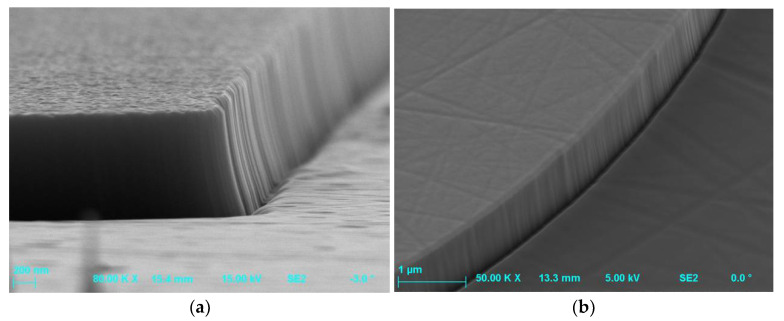
SEM profiles of SiC MESA structures with microtrenches (unevenness on walls of etched profiles) obtained after etching processes: (**a**) O_2_ flow rate = 2 sccm, SF_6_ flow rate = 20 sccm, *P_RIE_* = 100 W, *P_ICP_* = 1300 W, *p* = 5 mTorr, *t* = 12 min; (**b**) O_2_ flow rate = 50 sccm, SF_6_ flow rate = 50 sccm, *P_RIE_* = 50 W, *P_ICP_* = 2500 W, *p* = 7 mTorr, *t* = 5 min.

**Figure 5:**
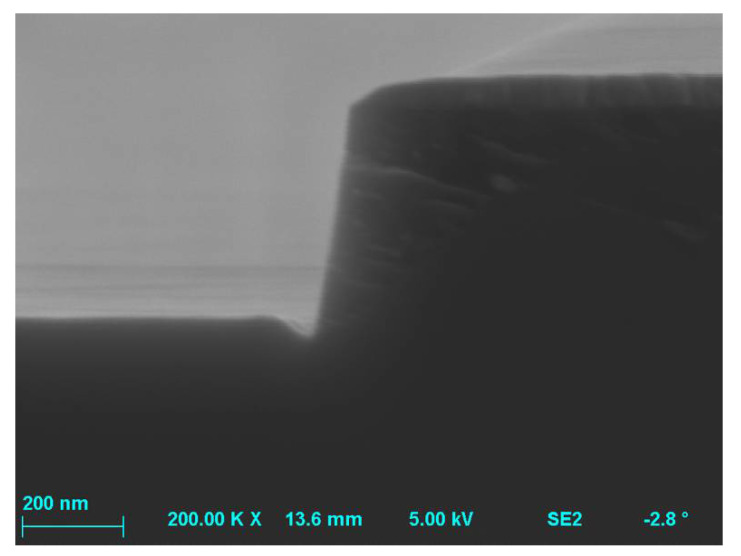
The SEM photo of the SiC/Cr MESA etched by SF_6_ + O_2_ plasma with a tip of the microtrench formation (at the bottom corner of the sidewall). The 65 nm-thick Cr layer is visible at the top of this MESA after the etching process with the following parameters: O_2_ flow rate = 2 sccm, SF_6_ flow rate = 20 sccm, *P_RIE_* = 100 W, *P_ICP_* = 1300 W, *p* = 10 mTorr, *t* = 10 min.

In the case of etching by ICP-RIE method of MESA or trench structures, there was also observed unevenness on the walls of the etched profiles, the so-called “microtrenches” [5,29,33,53,56]—in Figure 4. They appeared, for example, when a distance between electrodes in the reactor is reduced [53], or as a result of processes with dominant physical mechanisms of etching [6,56]. The formation of the microtrench may also be accompanied with the presence of the tip of the microtrench (Figure 5). This effect is due to locally high ion bombardment rate at the bottom corner of the sidewall, and thus exhibits a higher etch rate than a plane surface. The tip of the microtrench is very undesirable as it can cause locally high electric field and degrade the breakdown characteristics of power devices. The microtrenching can be avoided, e.g., by adding oxide to a Cl_2_-based plasma. In this case, the sidewall passivation oxide layer can be formed, reflecting incident ions away from the corner [5].

**Figure 6:**
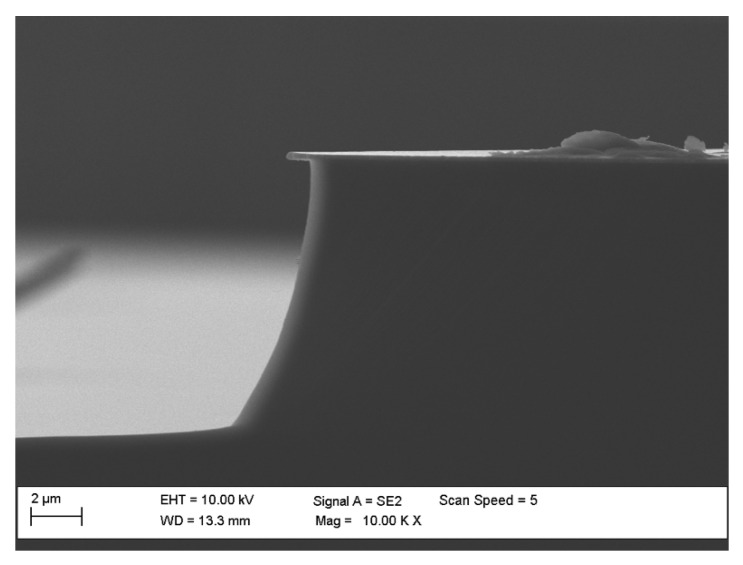
The edge SEM image of the SiC/Cr MESA structure with clearly pronounced Cr mask undercut after the etching process with the following parameters: SF_6_ flow rate = 100 sccm, *P_RIE_* = 25 W, *P_ICP_* = 2500 W, *p* = 7 mTorr, *t* = 119 min.

For the etching processes with a dominant chemical etching mechanism, i.e., when using high P_ICP_ source power, the effect of “undercutting” of the walls of the obtained structures was observed [6,29,56]—in Figure 6.

### 4.2. Micromasking and Mask Opening Width Effects

**Figure 7:**
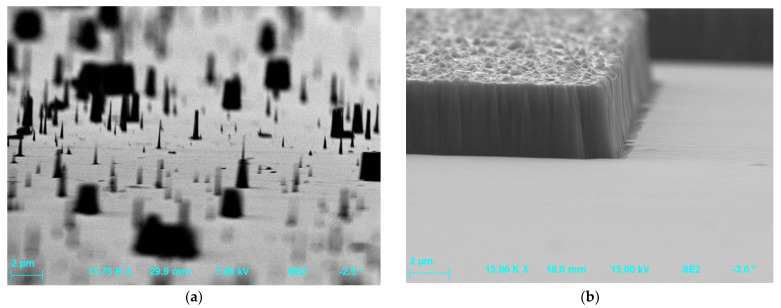
The micromasking effect observed in SiC etching processes: (**a**) with the use of the AZ 4562 resist and parameters: O_2_ flow rate = 2 sccm, SF_6_ flow rate = 20 sccm, *P_RIE_* = 100 W, *P_ICP_* = 2000 W, *p* = 7 mTorr, *t* = 1 min.; and (**b**) as the result of erosion of the aluminium mask (O_2_ flow rate = 2 sccm, SF_6_ flow rate = 20 sccm, *P_RIE_* = 100 W, *P_ICP_* = 900 W, *p* = 7 mTorr, *t* = 20 min).

During the etching process, as a result of the chemical reaction of plasma components with the etched surface, not only volatile compounds are formed, but also chemical compounds that can be re-deposited on the surface. Therefore, they can contribute to the occurrence of local heterogeneities in the chemical composition [34] and cause the effect of micromasking (Figure 7). In addition to non-volatile chemical compounds depositing on the etched surface, impurities present on the surface before the etching process (e.g., native oxides or dusts), or imperfections such as scratches of the surface may also be a source of micro-mask effect [12,21,60]. The phenomenon of micromasking was observed in the process of SiC etching as a result of aluminum mask erosion and formation of the non-volatile Al_2_O_3_ oxide [27]. The micro-mask effect was also observed for silicon, for which a very thin column structures (perpendicular to the etched surface) were formed during the etching process. In the literature, these structures are referred to “grass-like-structure” or “black silicon structure” [60]. By “black silicon structure” is meant a structure whose reflectance is close to zero [12].

Joo et al. in their paper [12] presented that the conditions of the plasma etching process, which produce the “black silicon structure” can be used in the process of controlled change of SiC surface roughness. This process is carried out by etching the Si/SiC structure in plasma, produced by depositing the Si overlayer (thickness of ~1 µm) on the SiC substrate, until the surface layer is completely etched. The Si layer is designated to initiate and restore the morphology of the “black silicon structure” on SiC, resulting in surface roughness. The degree of surface roughness of SiC can then be controlled by the duration of the etching process. The longer the etch time in plasma, the smoother the SiC surface.

Tanaka et al. [1] observed that addition of oxygen to the SF_6_ plasma may have the side effect of erosion of the metallic (nickel) mask used in the process, leading to the micro-mask effect, i.e., the formation of undesirable “grass-like” structures at the bottom of the etched profiles. Ni mask erosion was also noted by Li et al. [17] who investigated the trenches obtained in the process of etching of SiC with SF_6_ + O_2_ plasma with the use of various masks including SiO_2_, Ni, Ni/SiO_2_ and Ni/Al_2_O_3_. Microscopic (AFM) and spectroscopic (XPS) investigations of these structures’ surfaces after etching proved that the Ni/Al_2_O_3_ mask turned out to be the best one. For this mask, a trench structure was obtained with almost rectangular profile of the sidewall and without traces of metallic contamination. It is worth mentioning that the MOS structure made on a SiC sample etched with a Ni/Al_2_O_3_ mask [17] showed the best electrical properties, including critical breakdown electric field of 7.7 MV/cm. This value was much higher compared to the electrical properties of this type of MOS structures made on SiC samples etched with three other masks (SiO_2_, Ni, Ni/SiO_2_).

The micromasking effect may also occur when the etching process takes place under elevated pressure in the reactor chamber. Ekinci et al. [22] investigated the effect of process pressure on the etching rate and the morphology of 4H-SiC surface after etching in Cl_2_ + Ar + BCl_3_ plasma. The authors noticed that as the pressure in chamber increased, the etching rate decreased. The surface roughness of the etched samples also increased. The increase in pressure decreased the mean free path of interaction of plasma “particles” and increased the probability of their collisions, which in turn led to a decrease in plasma density. Taking into account the lower plasma density and the high SiC bonding energy, the etching rate tended to decrease. As the pressure increased from 8 mTorr to 30 mTorr, the etching rate decreased monotonically, which could be related to a decrease in the flux of reactive ions reaching the surface of the etched samples and/or a decrease in the amount of reactive Cl- ions in the plasma. The observed increase in surface roughness of etched samples, with increasing the pressure could, however, result from a decrease in the amount of reactive ions (with reduced kinetic energy as a result of ion–ion, ion–radical or ion–electron collisions) reaching the sample surface and slowing down the desorption process at the surface. Ekinci et al. [22] also noticed that addition of BCl_3_ to the Cl_2_ + Ar plasma improves the SiC post-etching surface morphology by reducing the surface roughness.

For SiC samples etched by ICP-RIE method with use of the Cl_2_ + O_2_ plasma it was observed that the addition of oxygen in the plasma may contribute to a change of the sidewall slant angle of etched structures and to the micromasking effect [5]. Tseng et al. presented in their paper [5] that the result of etching processes was also influenced by silicon or SiO_2_-coated silicon substrates which were used in the etching process as load wafers for transferring SiC samples to the process chamber. The use of these load wafers for a Cl_2_ + O_2_ plasma etching, in particular silicon wafer, promoted the micromasking effect. When using a silicon load wafer, the formation of SiC micropillars and SiO*_x_* micromasks was noted on the etched SiC samples. When using the SiO_2_-coated load wafer, the SiO*_x_* micromasking effect on SiC samples was still observed, but it was significantly reduced.

The influence of the width of the metal mask used in the etching process on the intensity of the micromasking effect, the so-called “mask opening width effect” was also reported in the literature [1,5]. Tanaka et al. [1] observed spike-shaped residues on the etched bottom surface of SiC trenches after SF_6_ + O_2_ etching and for the Ni mask opening width *x* in the range: 100 µm < *x* < 1 mm. The SiC trenches with a small pattern width were less susceptible to micromasking than those with a large pattern width [1,5], but an exception to this rule can be also found in the literature. The micromasking effect was observed by Tseng et al. [5] even for “narrow” trench structures, ~6 µm wide, after SiC etching in the Cl_2_ + O_2_ plasma. In the paper [1], the occurrence of the micromasking effect is explained by the balance of several factors, such as: the amount of reaction products, the ability to re-deposition on the etched side walls, the rate of evacuation of the reaction products and the mask opening width. In etching processes, there may be trench widths at which the first three above-mentioned factors balanced out, and the re-deposition of the reaction products on the etched bottom surfaces may occur when the mask opening width is between the balanced widths.

**Figure 8:**
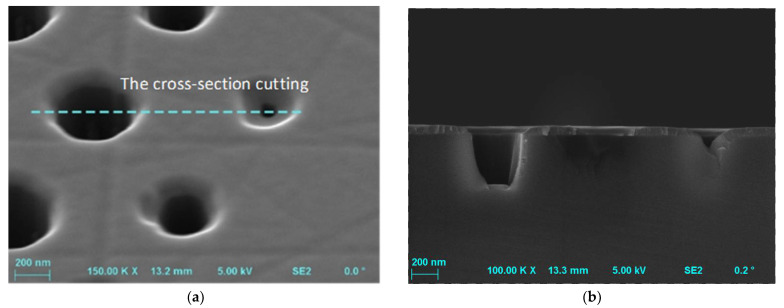
(**a**) The pattern after SiC etching process with parameters: O_2_ flow rate = 2 sccm, SF_6_ flow rate = 20 sccm, *P_RIE_* = 100 W, *P_ICP_* = 1300 W, *p* = 10 mTorr, *t* = 10 min. (**b**) The cross-section of the pattern shown in (**a**). The relationship between the mask opening width and the etched depth is visible, thus confirms the microloading effect. Designed diameters were 420 nm and 220 nm for indicated in (**a**) left and right holes, respectively.

The mask opening width used in the etching process also affects the depth of the etched structures, which is known as the so-called “microloading effect”. This effect can be defined as a decrease in the etch depth together with a decrease in the mask opening width. The reason for this may be the narrow mask opening preventing the penetration of ions and diffusion of the reaction products [1]. Figure 8 presents the SEM photo, which shows the relationship between the mask opening width and the etched depths, and thus confirms the microloading effect.

It is obvious that the micromasking effect, which significantly deteriorates the surface quality of etched structures, is particularly undesirable in the construction of electronic devices. Hence, it is so important to select the appropriate parameters of the etching process to eliminate the formation of residual structures. The risk of their formation can be eliminated, for example, by increasing the pumping rate of the chamber, increasing the temperature of the etched sample, or by gradually changing the etching conditions according to the etching depth obtained [1,40].

## 5. Experimental Verification of Some Conclusions on ICP-RIE Etching of SiC and Some New Results

**Figure 9:**
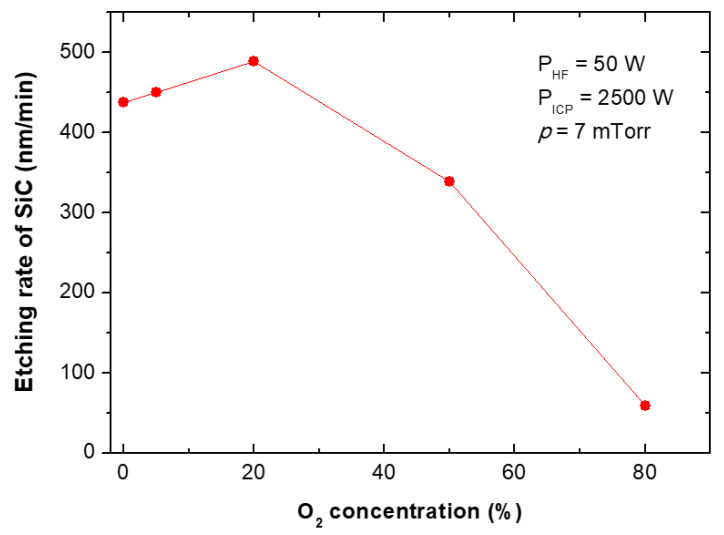
The relationship between oxygen concentration in the SF_6_ plasma and SiC etching rate. Fixed parameters are included in the inset.

**Figure 10:**
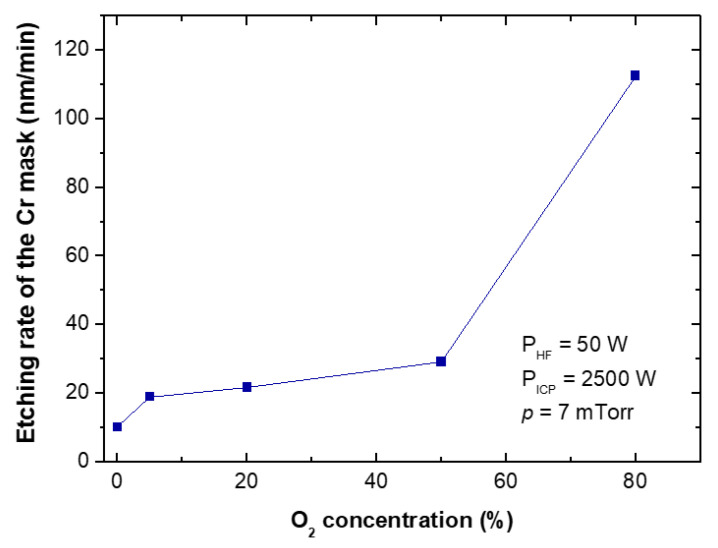
The relationship between oxygen concentration in the SF_6_ plasma and the Cr mask etching rate. Fixed parameters are included in the inset.

**Figure 11:**
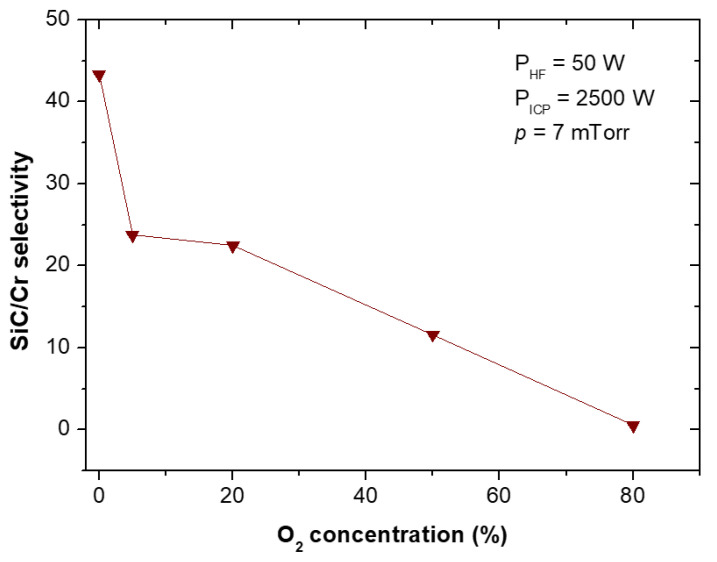
SiC/Cr selectivity vs. oxygen concentration in the SF_6_ plasma. Fixed parameters are included in the inset.

**Figure 12:**
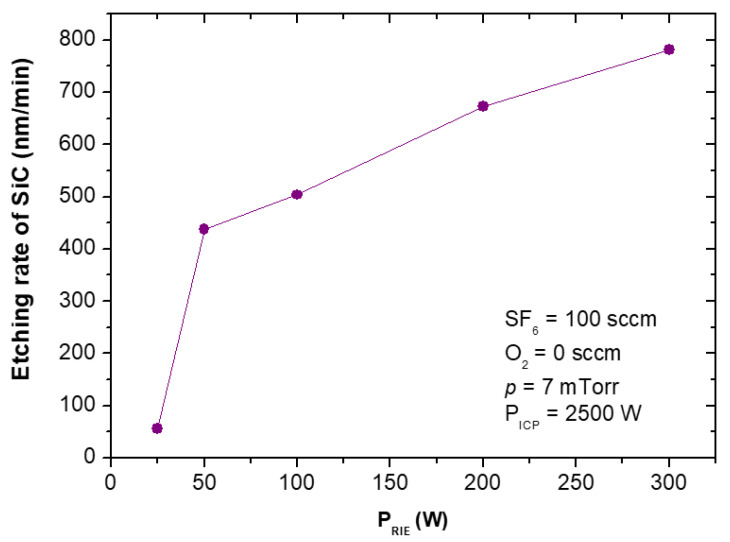
SiC etching rate as a function of RIE power applied. Fixed parameters are included in the inset.

**Figure 13:**
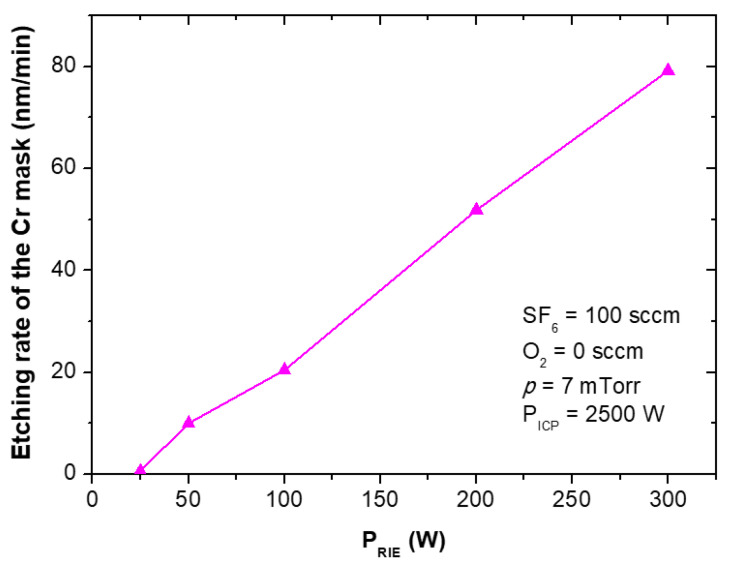
Cr mask etching rate as a function of RIE power applied. Fixed parameters are included in the inset.

**Figure 14:**
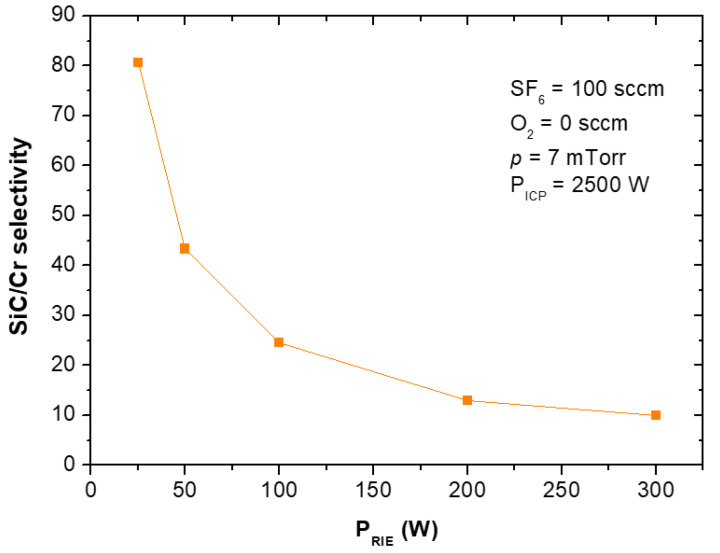
SiC/Cr selectivity as a function of RIE power applied. Fixed parameters are included in the inset.

**Figure 15:**
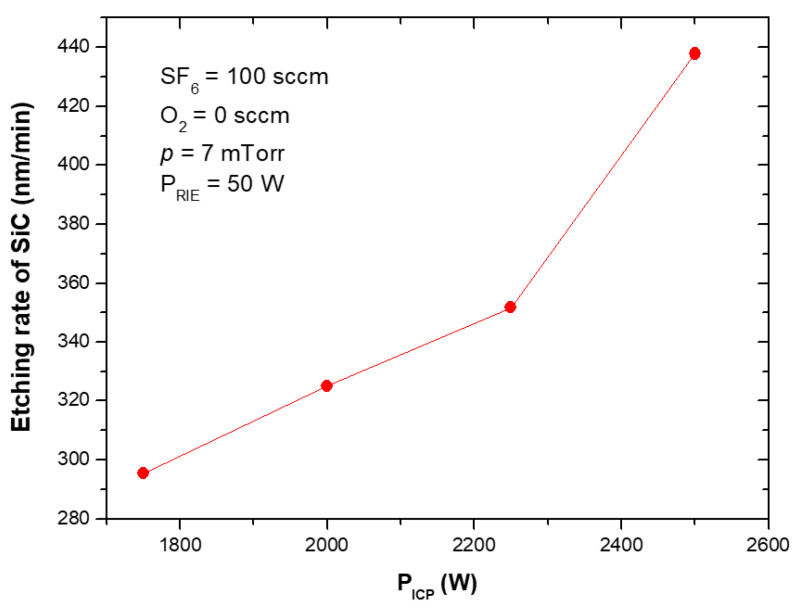
SiC etching rate as a function of ICP power applied. Fixed parameters are included in the inset.

**Figure 16:**
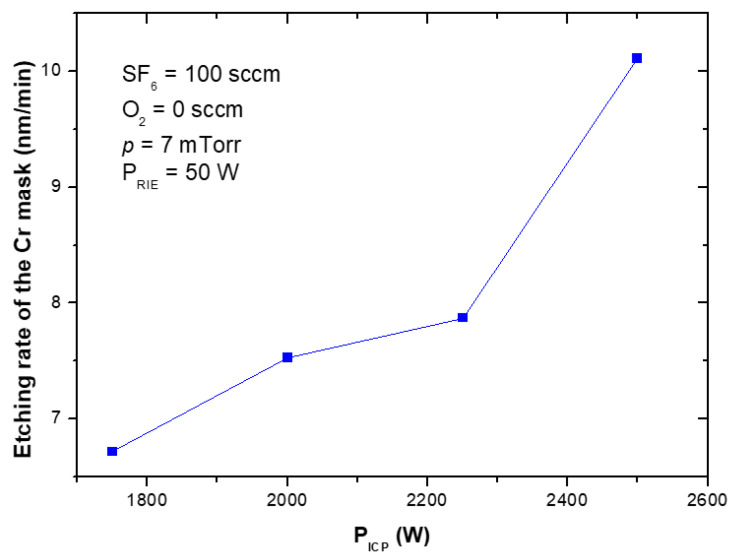
Cr mask etching rate of as a function of ICP power applied. Fixed parameters are included in the inset.

**Figure 17:**
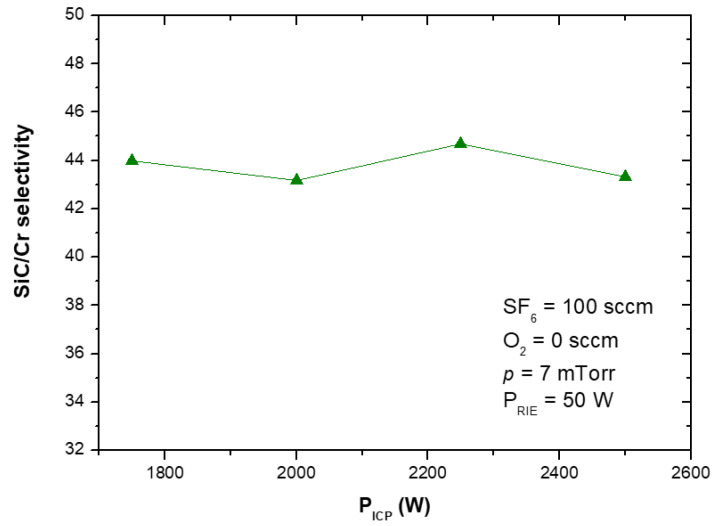
SiC/Cr selectivity as a function of ICP power applied. Fixed parameters are included in the inset.

The etching rates and selectivities of SiC/Cr etching were investigated under various etching conditions. The etching rates of SiC and chromium mask, as well as, SiC/Cr selectivities were studied as a function of: (i) oxygen concentration (Figure 9, Figure 10 and Figure 11), (ii) applied RIE power (Figure 12, Figure 13 and Figure 14) and (iii) ICP power (Figure 15, Figure 16 and Figure 17). Selected experimental results obtained in this study are presented for the first time in the literature. In our ICP-RIE processes, the etching time was determined by the total etch time of the Cr mask.

Although SiC plasma etching with a SF_6_ + O_2_ gas mixture has been reported in many papers (see Section 3.1 of this article), there is no information on the effect of the addition of O_2_ to the SF_6_ plasma on the etching rate of the Cr mask used in processes, and on the selectivity of SiC/Cr etching. In this paper, we report such etching characteristics for the first time—in Figure 10 and Figure 11, respectively.

The characteristic of the SiC etching rate as a function of O_2_ in SF_6_ + O_2_ gas mixture obtained in our research (Figure 9) is consistent with those presented in literature [7,23,30,42,44]. Many reports indicate that SF_6_/O_2_ = 4:1 (20% O_2_) is required to obtain the best SiC etching rate in ICP [7,23,30,42,44]. Our results are consistent with literature reports, where the SiC etching rate with SF_6_ + O_2_ plasma increases slightly, reaches its maximum at around 20% of O_2_, and then decreases (probably due to a dilution effect) [30]. From our experiments it can also be concluded that the SiC etching rate in SF_6_ + O_2_ plasma can be increased by more than 10 % with optimal oxygen content of 20% compared to SiC-etching in pure SF_6_ plasma under similar conditions (Figure 9). The highest SiC etching rate ~487 nm/min was achieved in this study for 20% of O_2_ and for high gas flow: 20 sccm O_2_ and 80 sccm SF_6_. This SiC etching rate value is very closed to the SiC etching rate of ~500 nm/min reported by Jiang et al. [44] for etching in SF_6_/O_2_/Ar gas mixture with ~30% Ar as optimal concentration. It is also worth mentioning that when etching of SiC in a different plasma—C_2_F_6_ + O_2_—it was shown that the etching rate increases with the addition of more O_2_, reaches its maximum at around 60% of O_2_, and then decreases [52]. On the other hand, only a slight and monotonical increase in SiC etching rate was observed at 40% of O_2_ content by Sugiura et al. [41] for SiC etching in the CF_4_ + O_2_ mixture.

The increase in the etching rate of Cr mask with O_2_ content in SF_6_ + O_2_ gas mixture was observed (Figure 10), while the SiC/Cr selectivity showed a decrease with increasing oxygen content (Figure 11). In Figure 11 the best SiC/Cr selectivity (43) is observed for pure SF_6_. It is possible that increasing the amount of oxygen in the plasma causes an increase in chemical reactions—mainly between Cr and O_2_—and the formation of volatile etch products of Cr-O type, which are removed very quickly at our high flow rates. Jiang et al. [42] found that the relative concentration of reactive F ions decreases with increasing O_2_ in the gas mixture, so the reaction between F and SiC is limited by gas phase reactions. It can therefore be assumed that the dominant chemical reaction in the etching of the Cr mask occurs between oxygen and chromium. Different flow rates can affect the residence time of the reactive radicals on the etched surface as well as the rate of removal of volatile etch products. Higher flow rates assist in the rapid removal of such products [42,44].

Figure 12 and Figure 15 show that the SiC etching rate in the SF_6_ plasma can be improved by increasing the applied RIE power or ICP power, and it increases with them. Similar trends in the dependence of the SiC etching rate on the applied power have been reported in literature, e.g., for SF_6_ + O_2_, C_2_F_6_ + O_2_ or SF_6_ + He gas mixtures [7,22,30,41,42,44,52]. It should be noticed that in case of relationship between the SiC etching rate and ICP power, in literature have been reported results for lower ICP power range, i.e., below 1000 W [22,30,44,52]. Our investigations presented in this paper extend this ICP power range considerably up to 2500 W.

It can be explained that an increase in the RIE power enhances the effect of physical ion sputtering on the etched surface and thus promotes the etching rate (Figure 12), while the increase in the ICP power increases the density of reactive ions in the chamber, which may result in the increase in chemical reactions on the etched surface and thus the etching rate (Figure 15) [22,30,42,44]. In Figure 12 it can be seen that there is a threshold energy (the dependence of the etching rate does not pass through the origin) that must be reached before etching begins [22].

In this paper, for the first time, we report the influence of both the applied power (RIE and ICP) on the etching rate of the Cr mask used in processes (Figure 13 and Figure 16), and on the selectivity of SiC/Cr etching (Figure 14 and Figure 17). The increase in both Cr mask etching rate and SiC etching rate with increasing the RIE power (Figure 13 and Figure 12) can be explained that with higher RIE power the mean ion energy was higher, and therefore the Cr mask and SiC sputtering were more likely. Similar upward trends are observed for the Cr mask etching rate and SiC etching rate with the increase in the ICP power (Figure 15 and Figure 16), which may be related to the increased density of reactive ions in the chamber and, consequently, the increased chemical reactions on etched surfaces.

**Figure 18:**
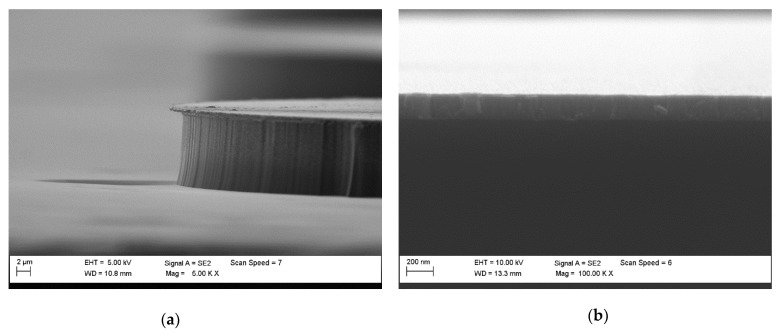
(**a**) The SEM photo of the SiC/Cr MESA structure with (**b**) ~210 nm layer of chromium at the top (the result of 119 min etching).

**Figure 19:**
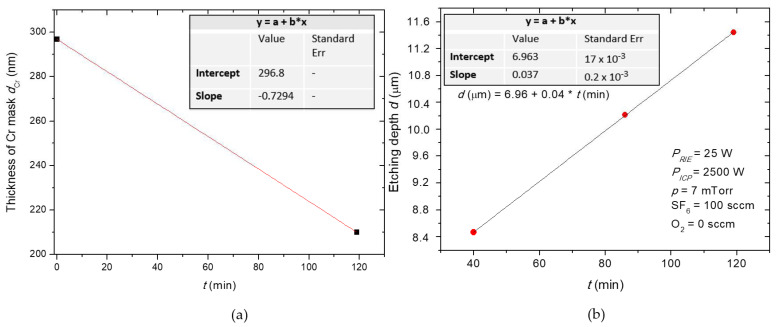
(**a**) The thickness of the Cr mask vs. time dependence used to estimate the time (*t* = 424 min) needed for completely etching of the Cr mask at the RIE power of 25 W. (**b**) The depth of SiC etching (with the Cr mask) vs. time (points) at three different stages of the etching process. From the linear fit of *d(t)*, the etching depth corresponding to a complete Cr mask removal was estimated at ~24 µm.

In Figure 14 is visible that SiC/Cr selectivity decreases exponentially with the increasing RIE power, and the best selectivity (~81) is observed at 25 W. It is worth noting that after the etching time of 119 min there was still a thick layer of Cr mask in etched MESA structures compared to the thickness of this mask (~297 nm) at the beginning of the etching process. Based on SEM measurements (Figure 18), the thickness of this Cr layer was estimated to be ~210 nm (Figure 18b). For RIE power of 25 W, the time needed to completely etch the Cr mask was very long and was estimated to be 424 min (Figure 19a). Thus, the SiC etching depth calculated for this time was ~24 µm (Figure 19b). For ICP power, a slight change in the SiC/Cr selectivity (Figure 17) is observed in the range of 1750–2500 W and the best selectivity is ~45 at 2250 W.

## 6. Conclusions

The etching of SiC by ICP-RIE method is influenced by many factors, which makes it difficult to achieve the desired results. Obtaining a fully repeatable and stable technology of the SiC etching with ICP-RIE method is associated with determination and optimization of process parameters which have a significant impact, among others, on the etching rate, surface morphology, sidewall inclination angle of MESA or trench structures, and the smoothness of the walls. Moreover, these parameters must not cause erosion of the mask material (i.e., not induce the phenomenon of micromasking), and also control a degree of the surface damage. These include the etching gas used (which may also be a multi-gas mixture), working gas flow, pressure in the reactor chamber, glow discharge excitation parameters (RF power applied), surface area of the etched material and the substrate temperature.

It is also important to find a balance between the physical and chemical mechanisms of the etching. This allows, among others, to optimize the anisotropy of etched patterns. The idea is to skillfully “control” the flux and energy of reactive ions to avoid sidewalls unevenness (i.e., phenomenon of microtrenching when the physical etching mechanism is dominant) and undercutting of sidewalls (when the chemical etching mechanism is dominant). The selection of appropriate etching conditions will allow for etching specific patterns in SiC with the accuracy required for applications in semiconductor devices technology. It should be noted that the control of the SiC etching process is necessary in extending the range of SiC applications in various electronic and optoelectronic devices.

The original results of dry etching with the use of chrome mask presented in Section 5 have been developed with the intention of producing of power devices in silicon carbide technology. The conducted research fully confirms the usefulness of the technology in the development of power devices for advanced power electronics. The dynamically growing production of micro- and nano- electromechanical systems (MEMS/NEMS) is a new challenge in the development of dry etching process due to higher etching rate while maintaining surface quality. For this reason, factors influencing the etching rate and surface quality are nowadays widely analyzed, including the content of oxygen and argon in the fluorine plasma, the composition of the fluorine plasma itself, and recently also the substrate temperature and other factors. The possibilities of using alternative masking materials other than copper (which is the superior masking material in terms of selectivity) are also being explored to ensure better manufacturing compatibility while improving the selectivity needed for very deep trenching. In deep trenching process, it is important to analyze all performance limiting phenomena resulting from the sputtering and re-deposition of masking material such as microtrenching and pillar formation. While in the technology of semiconductor devices some phenomena are to be ignored, in technologies focused on high-aspect-ratio shapes the issues of selection and treatment of masking material become critical for many applications.

## Figures and Tables

**Table 1:** Various plasmas used in the ICP-RIE processes and the main experimental aspects (slant angle, etching rate, and surface morphology) of the SiC etching.

Plasma	Slant Angle Ref.	Etching Rate Ref.	Surface Morphology Ref.	All References
SF_6_	[23,30]	[21,28,37,38]	[8,18,21,28,30,38]	[8,18,21,23,28,30,37,38]
SF_6_ + O_2_	[7,17,23,26,29,30,35,39,40]	[1,7,23,25,26,27,30,35,38,39,41,42,43,44,45,46,47]	[1,4,12,17,20,23,27,29,30,38,39,42,43,44,45,46]	[1,4,7,12,17,20,23,25,26,27,29,30,35,38,39,40,41,42,43,44,45,46,47]
SF_6_ + Cl_2_	-	[38]	[38]	[38]
SF_6_ + O_2_ + Ar	-	[44,48]	[44,48]	[44,48]
SF_6_ + Ar	-	[37,38]	[37]	[37,38]
SF_6_ + He	-	[21,41]	[21]	[21], Ref. in [35,41]
SF_6_ + CH_4_	[30]	[30]	-	[30]
SF_6_ + CH_4_ + He	-	[21]	[21]	[21]
CHF_3_	-	[49]	[49]	[49], Ref. in [50]
CHF_3_ + O_2_	[35]	[35,36]	[49]	[35,36,49]
N_2_	-	-	[13]	[13]
O_2_	-	-	[13]	[13]
Ar	-	[41]	[44,51]	Ref. in [35,41,44,51]
C_2_F_6_	[52]	[52,53]	[32,52]	Ref. in [30,32,52,53]
C_2_F_6_ + O_2_	[52,53]	[52,53]	[32,52,53]	[32,52,53]
NF_3_	[54,55]	[54]	[54,55]	Ref. in [30], Ref. in [35,54,55]
NF_3_ + O_2_	-	[56]	[54,56]	[54,56]
NF_3_ + Ar	-	[56]	[56]	[56]
NF_3_ + CH_4_	[54,55]	[50,54]	[50,54,55]	[50,54,55]
CBrF_3_ + O_2_	[35]	[35]	-	[35]
CF_4_	-	[21,41]	[21]	[21], Ref. in [30], Ref. in [35,41]
CF_4_ + O_2_	[33]	[33,41,51]	[33]	[33], Ref. in [35,41,51]
CF_4_ + He	-	[21]	-	[21]
CF_4_ + Ar	-	[51]	-	[51]
CF_4_ + Cl_2_ + O_2_	-	-	[11]	[11]
BCl_3_	[23]	[22]	[22]	[22,23]
BCl_3_ + N_2_	[23]	[23]	-	[23]
BCl_3_ + Cl_2_	[23]	[23,57]	[57]	[23,57]
BCl_3_ + Ar + Cl_2_	-	[22,57]	[22,57]	[22,57]
Cl_2_	[23]	-	[23]	[23]
Cl_2_ + Ar	-	[38]	[4,19,38]	[4,19,38]
Cl_2_ + O_2_	[5]	[5]	[5,23]	[5,23]
